# Facilitating Children’s Club-Organized Sports Participation: Person–Environment Misfits Experienced by Parents from Low-Income Families

**DOI:** 10.3390/children9111746

**Published:** 2022-11-14

**Authors:** Lonneke van Leeuwen, Anne Annink, Kirsten Visser, Marielle Jambroes

**Affiliations:** 1 Public Health Department, Julius Center for Health Sciences and Primary Care, University Medical Center Utrecht, Universiteitsweg 100, 3584 CG Utrecht, The Netherlands; 2 Utrecht University School of Governance, Utrecht University, Bijlhouwerstraat 6, 3511 ZC Utrecht, The Netherlands; 3 Department of Human Geography and Spatial Planning, Faculty of Geosciences, Utrecht University, Princetonlaan 8a, 3584 CB Utrecht, The Netherlands

**Keywords:** club-organized sports, children, low-income families, parents, socioecological model, person–environment misfit

## Abstract

Despite the many benefits of club-organized sports participation for children, studies have shown that sports participation is lower among children from low-income families than among children from middle- or high-income families. Adopting a socioecological perspective, the main aim of our study was to identify and describe experiences of person–environment (PE) misfits in relation to parental facilitation of children’s sports participation. We conducted 24 interviews with parents from low-income families. PE misfits were found in multiple behaviors related to the facilitation of children’s sports participation: financing sports participation; planning and investing time; transporting children; acquiring, processing, and providing information; and arranging support. Across these PE misfits, influential attributes were found on the individual level (e.g., skills) as well as within the social, policy, physical, and information environment. In response to PE misfits experienced, parents deployed multiple strategies to reduce these PE misfits, aimed at enhancing either themselves (e.g., increasing financial capacities) or their environments (e.g., arranging social support). These results provide an insight into experienced PE misfits that took the form of multiple specific behaviors which parents found difficult while facilitating their children’s sports participation. Furthermore, the results provide insight into the environmental and individual attributes that were involved in these PE misfits, and into how parents modified themselves or their environments in order to make their environments more supportive. The study contributes to future research on individual and environmental influences on parental facilitation of their children’s sports participation, as well as on the development of multilevel interventions aimed at increasing sports participation among children from low-income families.

## 1. Introduction

It is widely known that children benefit from club-organized sports participation (hereafter: sports participation). Sports participation by children contributes to children’s overall level of physical activity [1] and thereby to physical benefits, such as a healthy vascular system and maintenance of a healthy bodyweight [2]. In addition, sports participation has been shown to have psychological benefits for children such as emotional self-efficacy and fewer depressive symptoms, as well as social benefits such as social integration and social well-being [3].

Despite the many benefits of sports participation for children, studies in multiple countries have shown that sports participation is lower among children from low-income families compared to children from middle- or high-income families (e.g., [4,5,6,7]). This inequality is also observed in The Netherlands. In 2017, 83% of children (aged 12–16) from affluent families were sports-club members, whereas the corresponding percentage for children from medium- and low-affluence families was 67% and 46%, respectively [8]. This inequality does not appear to be explained by lower motivation for sports participation among children from low-income families in The Netherlands: three out of four children from low-income families who do not participate in sport expressed at least some level of desire to do so [9].

Parents, or children’s other main caregivers (hereafter: parents), play an important role in facilitating their children’s sports participation [3]. However, most studies to date have focused on the role of child-related factors to understand associations between income and sports participation [10], such as the role of children’s perceptions about the sports behavior of family and friends [11] or their perceptions about parental support for sports participation [12]. Relatively few studies have focused on understanding factors that influence the degree to which parents from low-income families are able to facilitate their children’s sports participation. These few studies have shown that low-income parents experience a number of barriers to facilitating their children’s sports participation. First, parents reported experiencing issues with time and scheduling [13,14,15], because of, for example, work, being a single parent, or having multiple children. Second, they reported issues with transportation [13]. Third, they experienced financial barriers such as costs associated with sports participation [13,14,16] or a lack of knowledge about financial support options, or they experienced difficulties with continuously having to apply for financial support for their children’s sports participation [15].

To increase sports participation among children from low-income families, it is essential to increase the knowledge about the individual and environmental factors that impede or support their parents’ facilitation of sports participation. Therefore, using a socioecological perspective, this study aims to contribute to the current knowledge about relevant factors.

### 1.1. Theoretical Background

Socioecological theories suggest that behavior is influenced by multiple factors on both the environmental and the individual level [17,18]. The environmental level includes influential attributes of environmental domains such as the social (e.g., behavior of friends), physical (e.g., built environment), information (e.g., media), policy (e.g., rules and regulations), and economic environment (e.g., costs). The individual level includes demographic, biological, and psychosocial attributes (e.g., needs and capabilities). In addition, it includes environmental perceptions, such as the perceived safety, attractiveness, or accessibility of the (e.g., physical) environment.

Socioecological theories further suggest that any behavior represents a joint influence of both individual and environmental attributes [19]. These joint influences may take the form of person–environment (PE) (mis)fits. A PE misfit is a mismatch between individual and environmental attributes, leading to individuals not being able to behave or live in accordance with their preferences and plans. This, in turn, may lead to negative reactions such as discomfort, withdrawal, and stress. A PE fit is the opposite of a PE misfit: individual and environmental attributes match, and individuals can behave or live in accordance with their preferences and plans [19]. Individuals may strive to achieve higher levels of PE fit, either by enhancing themselves or by enhancing their environment [19,20]. This process has also been referred to as human–environment optimization or active coping [21].

In the last decades, the number of studies on PE (mis)fit have increased rapidly, and fit theories have been developed in multiple fields, including the fields of health and stress, and organizational psychology (where it is referred to as, e.g., person–job fit or person–supervisor fit) [22]. The PE (mis)fit concept has also been applied in socioecological studies on physical activity. For example, a qualitative study among older public housing tenants aimed to better understand the joint influence of individual and environmental factors associated with the tenants’ ability to be physically active in their residential environment [23]. It found that multiple PE fits, which encompassed the fit between the tenants’ capacities (e.g., physical) and opportunities to be physically active in their residential environment, were fundamental to the tenants’ physical activity.

The previously mentioned studies that explored influences on facilitation of children’s sports participation by parents from low-income families [13,14,15,16] provide important insights into the relevant individual and environmental attributes. In line with the studies of Rodrigues [13] and Holt [14], we believe that a socioecological approach is necessary to better understand the degree to which parents from low-income families are able to facilitate their children’s sports participation. More specifically, we believe that the PE (mis)fit concept may add to this understanding. First, it adds to the current knowledge by elucidating how individual and environment attributes jointly influence parental facilitation. Second, it adds to an understanding of the strategies that parents use to reduce levels of PE misfit. Consequently, these insights provide a valuable basis for the development of multilevel interventions that aim to enhance specific PE fits, which should be more effective than single-level interventions [17,19]. Insights into parents’ own strategies to reduce PE misfit provide promising entry points for such interventions, as these may support such coping strategies.

### 1.2. Aims of the Study

The first aim of the study is to identify and describe experiences of PE misfits, and related environmental and individual attributes, during the facilitation of children’s sports participation by parents from low-income families. The second is to describe how parents deal with PE misfits or (aim to) reduce PE misfits. The third is to describe experiences of parents who have experienced less misfit than their counterparts. These experiences may serve as positive examples providing insight into individual and environmental attributes contributing to PE fit.

## 2. Materials and Methods

### 2.1. Design

The current study is based on semi-structured interviews with parents from low-income families. A qualitative approach was chosen to obtain insight into participants’ experiences with the facilitation of their children’s sports participation, with a focus on exploring experienced PE misfits.

The current study was part of the transdisciplinary study Vital@2040, which focuses on increasing sports and physical activity among children at risk of decreased sports participation and physical activity. 

### 2.2. Procedure

Parents from low-income families were recruited via diverse strategies. Most parents/parental couples were recruited via two charitable organizations that provide financial support for children’s sports participation and, more generally, children’s participation in society (18/24 interviews). Parental applicants for financial support were approached and briefly informed about the study by volunteers working for the charitable organizations. If applicants were interested in participating, they were asked to give their consent for the transfer of their contact information from the charitable organization to the researchers. In addition, three parents were recruited by the first author and an expert by experience in the courtyard of a food bank. Two parents were recruited via the researchers’ professional network. One parent signed up for the study after reading about it in a newsletter about the U-pass (city passes). These additional participants all possessed U-passes, which indicated that their families were qualified to receive financial support from the municipality for sports and cultural activities. The charitable organizations as well as the U-pass focus on financially supporting local families with ≤125% of the Dutch minimum income. All recruitment took place in the Dutch cities of Eindhoven and Utrecht.

Prospective participants were contacted by one of the researchers to schedule a 15-min telephone conversation. During this conversation, the researcher explained the study and procedures in detail, in line with the content of an information letter. If the prospective participant re-confirmed the desire to participate in the study, an appointment was scheduled for an interview. Participants were offered the choice of a face-to-face interview or an online video conference. All prospective participants confirmed that they were able to read information in Dutch. The information letter was sent to the participants by email after the phone conversation, requesting that they read the information letter prior to the interview.

During the interviews, participants were first given the opportunity to ask questions about the study. It was communicated (by letter and orally) that participants could withdraw from the study at any time, without having to provide a reason. Participants’ oral informed consent was collected and audiotaped.

To be able to describe the sample, information about each participant’s demographics was collected through an online survey completed by the interviewer, after which the interview commenced. Interviews were audiotaped and transcribed verbatim. Any information that might identify individuals was pseudonymized using a pseudonymization protocol [24]. Participants/couples received a EUR 15 gift voucher for their participation.

### 2.3. Materials

#### 2.3.1. Online Survey Demographics

The online survey consisted of four questions to collect data about the participant’s age (answer options: age categories, ranging from 18 to 50+), gender (answer options: female, male, not female/male, both female and male), level of education (answer options ranging from no education to academic education), and country of birth (answer options: The Netherlands and outside The Netherlands). The responses are presented for descriptive purposes only in Table S1 in Supplementary Materials.

#### 2.3.2. Interviews

Twenty-four interviews were conducted, of which 12 were held face-to-face and 12 were held online via video conferencing. The interviews commenced with questions about the children in the family/household (hereafter: family), e.g., number of children, their ages and genders, and whether they currently participated in sport. If during the interview references were made to whether the parent was a single parent or not, this was also recorded (see Table S1 in Supplementary Materials).

The interviews were structured around three sets of questions, probing for: (1) positive and negative thoughts or beliefs about sports for children and organizing children’s sports participation, (2) experienced or expected constraining and facilitating factors while/for facilitating children’s sports participation, and (3) suggestions for parent-targeted solutions to help increase sports participation among children from low-income families. Albeit using different formulations, the aim of these questions was to probe for experiences of PE (mis)fits. If not mentioned spontaneously, the interviewer probed for thoughts and experiences in relation to potential barriers such as transport, clothing, time, money, information, and support. In addition, a number of questions were asked for other research purposes [25] and to collect participants’ evaluations about the study (not reported in this article). Including these additional questions, interviews lasted between 29 and 87 min.

### 2.4. Participants

Of the 24 interviews, 22 were with individual parents and two were with parental couples. So, 26 parents were interviewed from 24 families, of which 22 participants identified as female and four as male. In relation to the child(ren)’s current sport status, 12 families included only children who participated in sports, 8 families included only children who did not participate in sports, and 4 families included both children who participated in sports and those who did not participate in sports. Of the 24 interviews, 13 were with participants living in Utrecht and 11 were with participants living in Eindhoven. More information about the participants—collected during the telephone conversation, the online survey, and the interview—is presented for illustrative purposes in Table S1 in Supplementary Materials.

The participants were considered as coming from low-income families given that they were recruited via charitable organizations that provide support for children from low-income families and/or participants told the researchers that they received support from these organizations. 

### 2.5. Data Analysis

The pseudonymized interview transcripts were analyzed using NVivo 12 software [26]. A naturalism research model was used, meaning that the interview data were treated as if they were giving direct access to participants’ experiences. The naturalism research model differs from, e.g., a constructionism research model, which focuses on understanding how social realities are constructed and sustained [27].

During the analyses of the transcripts, open and more directed approaches were combined. There was an openness to discovering diverse experiences of misfits and parental strategies to reduce misfits or deal with misfits. A more directed content analysis approach [28] was chosen based on socioecological theories. In practice, this means that these theories provided a framework for understanding the nature of the involved attributes in the misfit (individual and environmental), as well as subcategories in environmental factors (e.g., physical environment, social environment). Text fragments were first coded on misfit topics. Then, individual and environmental attributes involved in the misfit were coded, as well as parental strategies to deal with misfits. In addition to personal experiences, participants’ accounts of experiences of other low-income parents within their social environments (e.g., neighbors) were also coded. These second-hand experiences are reported in the Results section only if these experiences were also reported as personal experiences.

The first and second author each coded the same two interviews and discussed their codes and categorizations. Then, the first author coded the remaining interviews. Guided by previous comparable research among parents from 17 low-income families [14] combined with a review on sample sizes for data saturation [29], it was expected that for this rather homogenous study sample (low-income parents living in the Netherlands recruited mainly by charitable organizations), around 20 interviews would be sufficient to reach data saturation. After 17 interviews, 95% of the codes of the final code list were created, indicating a high level of data saturation after having analyzed 24 interviews.

In the description of the results, non-specific forms of semi-quantification (e.g., ‘multiple’, ‘a few’) are used to convey general patterns within the data [30]. As with any qualitative research, empirical findings cannot be generalized beyond the study population, which is also outside the scope of qualitative research.

## 3. Results

PE misfits were identified in relation to the following parental facilitating behaviors: financing sports participation; planning and investing time; transporting children; acquiring, processing, and providing information; and arranging support.

In the following sections, the environmental and individual attributes involved in these misfits are described, as well as the strategies that participants deployed to reduce or deal with the misfits. Last, the experiences of participants whose comments indicated that they experienced no or little misfit in relation to these behaviors are described.

### 3.1. Financing Sports Participation

Multiple participants stated that financing children’s sports participation was difficult, not an easy task, had not always been possible, and that they had to put effort into preparing and organizing their finances to be able to finance their child’s sports participation. These participants perceived the financial subvention from the municipality for children’s sports participation as low, whereas children’s sports participation was associated with (high) costs: sports membership/contribution, (multiple sets of) sports clothing for either practice or matches, necessary accessories (e.g., hockey sticks), replacement of clothing/accessories when outgrown/broken, traveling expenses to and from sports clubs for practice and matches, snacks at the sports club, and children’s attendance at extra-curricular activities organized by the sports club, such as camps. Participants were struggling with these costs, which were burdening their already tight budget:

“Clothing and shoes. Two T-shirts, two shorts. …[My child] got the accessory he needed for sport from [aunt/uncle]. That was hard for me to buy. …Because we have little money. …Currently, I am on benefit and it’s really little [money] to live on. Just for food. So, with sport comes extra costs. (Interview 18).”

Multiple participants stated that their current financial resources were limited. Reasons for limited financial resources included being on benefit or under financial administration, having to pay for children’s swimming lessons, having multiple children who (want to) play sports, and having no or a low-income job. Last, the absence of a co-financing partner was given as a reason:

“They [the co-parents of participant’s children] told me: you take care of it [buy a sport accessory]. I think: well, I’m not going to do that [by myself], but in the end, that negatively affects your children. …So, I am going to take care of it …by saving money on groceries and saving small amounts of money [to buy the sport accessory for the child]. (Interview 08).”

For this participant, the absence of a co-financing partner strained financial resources for sports participation. The participant’s strategy to enhance the financial fit—saving up money—seemed to be motivated by the expected negative impact of non-sports participation on this participant’s child. Other strategies to reduce or deal with misfit mentioned by participants were purchasing cheaper versions of sports clothing/accessories (e.g., secondhand clothes and/or accessories rather than new ones) or borrowing them, arranging and acquiring financial support for sports participation from funds, giving sports clothing or accessories to their children as presents (e.g., at birthdays), arranging or accepting financial support for sports participation from family members (see also the earlier quote from Interview 18), and living by a strict budget plan whereby future costs for sports participation are taken into account and financial choices are made in favor of sports participation.

Some participants, however, indicated that they experienced no or little difficulty in financing sports participation. This was mainly because they received financial support from funds in the municipality that made it possible for them to pay sports contribution fees and to purchase sports accessories, such as clothing. (More detailed results about individual and environmental attributes involved in acquiring financial support from funds are described elsewhere [25]).

“I cannot pay for [my children’s sports participation], but because of [fund] I can send my children there. I am really glad about that. (Interview 03).”

In addition, some sports club offered free clothing for matches, facilitated the hiring or purchasing of sports clothing or accessories at a discount, or arranged sponsors who contributed financially to these items. In addition, in some cases, the costs associated with their children’s sports participation were not that high, because of the low contribution fees or because no specific and expensive sports clothing or accessories were needed for the sport.

Collectively, the results show that participants experienced a misfit between the financial demands of sports participation on the one hand and their financial resources on the other hand. Environmental attributes involved in this misfit were high costs associated with sports participation and lack of financial support from the municipality. Individual attributes were limited financial resources as a result of, e.g., low income, being a single parent, and having multiple children. Participants deployed diverse strategies to reduce financial difficulties such as acquiring financial support and saving up money. Few financial difficulties were experienced by those who had children who participated in a non-expensive sport or encountered supportive policies within the municipality (availability of funds) or at the sports club (supportive policy for acquiring clothing/accessories) (environmental attributes).

### 3.2. Planning for, and Investing Time in, Children’s Sports Participation

Many participants expressed difficulties in finding the necessary time to facilitate their children’s sports participation. They stated that, in their experience, facilitating sports participation was associated with diverse time-demanding activities such as transporting children to and from the sports club for practice or for (away) matches and remaining at the sports club during practice and matches. The time investment and planning relating to sports participation was difficult to combine with other time-demanding activities, such as going to work:

“Practices are often during weekdays. I worked from 9 a.m. until 6 p.m. and by the time I got home it was almost 7 p.m. Then, there was absolutely no time to bring [my child] some place. (Interview 17).”

In addition to having to work, participants mentioned several other reasons why their resources in terms of time were limited. Reasons included bringing their children to swimming lessons, carrying out household tasks, preparing dinner, taking care of other/younger children in the family, and managing health issues of the child or of their own. In particular, single parents’ inability to share some of the burden with a partner/co-parent contributed to their struggling with finding the time to facilitate their child’s sports participation. Additionally, having multiple children participating in sports (on the same days but in different locations) limited their time available for investment in each of their children:

“In the beginning, [planning children’s sports participation] was really hard for me. [The children] had matches or practices around the same time and I had to remain [at the sports club] from 4 to 6 p.m. …Later, I let that go. I left them there and went home to make diner or shop for groceries. …Currently, they play sport on different days… so that’s no longer a problem. (Interview 11).”

This participant dealt with the time/planning misfit by re-allocating time from being at the sports club to preparing dinner, thereby increasing this parent’s time resources. In line with this, multiple participants stated that they actively managed their time to fit the child’s sports schedule into the (household) schedule. Other strategies included laying out the child’s sports clothes and accessories in the morning, sharing tasks with co-parents, and adapting normal routines in favor of sports participation, e.g., eating at a later time or not as a family.

Some participants seemed to experience no or little PE misfit in relation to time and planning. This resulted from having a co-parent with whom to share the tasks. Furthermore, many had prioritized their children’s sports participation and already actively managed their time, thereby potentially preventing a time/planning misfit:

“I am always planning as a single parent and working part-time during the day.… [The children] have to eat on time and they don’t play sport on the same day. If I have to take them somewhere, I make sure that my house is in order. So, the whole week I’m busy with them eating on time and making them ready [for sports participation]. …But I don’t experience it as a burden. (Interview 04).”

To summarize, a number of participants experienced a misfit between the time/schedule demands of sports participation on the one hand and their time resources on the other hand. Environmental attributes involved in this misfit were the time-demanding activities associated with sports participation, such as transport. Individual attributes were the participants’ involvement in other time-demanding activities such as work and family or household activities. Participants deployed diverse strategies to reduce misfit, including adapting their normal household routines. Misfit was experienced to a lesser extent by those who had a co-partner, prioritized sports participation, and actively managed their time (individual attributes).

### 3.3. Transporting Children between the Home and the Sports Club

Multiple participants stated that they experienced difficulties in covering the distance between the home and the children’s sports club or locations for extra activities related to the sport (e.g., extra trainings or matches). They referred mostly to the long distance between the house and the child’s sports club (or other clubs for away matches) or not having any sports clubs nearby, as illustrated by the following quote:

“All my nieces and nephews participate in sports. They all go there by walking or cycling, it’s all in the neighborhood. Then I think: Why isn’t [sports participation] organized like that [where I live]? …I think it’s way better organized there, by the municipality. (Interview 02).”

Additionally, the route was often considered too unsafe for the child to travel to the sports club independently (e.g., by bike). Children would have to travel in the dark or via roads with a lot of motor traffic, or would be confronted with unsafe social situations on the route, involving, e.g., drunk individuals or bullying peers. Last, participants lacked a social network that could support them in arranging transport.

Transport difficulties were experienced in particular by participants with children who relied on them as parents for transport, with no access to their own motor vehicle to cover the distance between the home and the sports club, or with a lack of time to transport the children (see Section 3.2). 

Strategies to reduce this misfit included borrowing a car, arranging or accepting support from other parents for co-traveling, using public transport (although this was referred to as a highly time-consuming means of transport), or only selecting a sports club nearby:

“When I saw the flyer [about the sports club], I saw that it was only a two-minute walk from my house. So, no transportation needed. [The children] can go there by themselves. That triggered us to go and see whether the children would like [the sport] and they are doing [this sport] since then without complaining. (Interview 12).”

Participants who could arrange co-traveling with others or were part of a supportive (online) social network with parents of their children’s teammates experienced no or little difficulty with transport. Often, this social network was initiated and facilitated by the sports club. Within these social networks, helping one another out and arranging co-traveling was very common:

“If it was an away game, [my partner] would make arrangements [with other parents] about who would travel together with whom in the cars.… Via a WhatsApp group … initiated by the sports club. The coaches and trainers also ask who wants to ride with them. (Interview 11).”

For other participants, the sports club was nearby, transport to cover the distance was available, or their children were able to cover the distance independently, e.g., by bike.

Overall, the results show that multiple participants experienced a misfit between the transport demands of children’s sports participation on the one hand, and their transport resources on the other hand. Environmental attributes involved in this PE misfit were too long or unsafe routes between the home and the sports club or lack of a social network. Individual attributes were lack of a motor vehicle and of time. Participants deployed diverse strategies to enhance PE fit, including arranging co-traveling. Less misfit was experienced by participants who had a supportive social network or a sports club nearby (environmental attributes). They had transport available or their children were able to travel to the sports club independently (individual attributes).

### 3.4. Acquiring, Processing, and Providing Information

Multiple participants described how they experienced difficulties in acquiring, processing, and providing the necessary information for organizing sports participation. This related to, e.g., acquiring information about available sports opportunities, how to register at the sports club, how to provide the information necessary for registration, and how to apply to charitable funds for financial support for sports participation.

In addition, there was too little information available to them about sports opportunities. They wished more information would be available and that more easily accessible and free options for children to sample (different) sports should be offered. Additionally, registration systems at sport clubs and procedures to acquire financial support from funds were perceived as complex. (More detailed results about perceptions relating to the procedures involved in acquiring financial support from funds are described elsewhere [25]).

A number of participants explained that they themselves or other low-income parents within their social environment had no computer, lower skills in reading and writing (in Dutch), and lower online media skills (e.g., writing emails or completing online forms). This played a role in their experiencing difficulties in acquiring, processing, and providing the necessary information for organizing sports participation:

“I said: “I don’t understand your website. There is so much information. I have trouble with registering [my child at your sports club]. And where do I find information about the contribution? Can you please explain [how that works]?” He said: “I am busy”. “Okay”, I said. “Thank you.” And then I left. My [child] said: “I don’t feel like playing sport there anymore.” (Interview 23).”

Previously, the participant had had quite a different experience at another sports club:

“The coordinator at the sports club explained to me in simple words: “Registration works like this.” He wrote it on a note for me, what I should do exactly. Easy. I did it immediately. (Interview 23).”

These quotes illustrate the finding that participants tried to arrange support from others to deal better with understanding and providing information necessary for their children’s sports participation. These others were working for the sports club, professionals from other organizations such as the child’s school or social organizations, or acquaintances/neighbors. The (un)supportive behavior by these others contributed (or did not contribute) to being able to deal with understanding and providing information.

Other participants did not experience any difficulties in relation to information because they actively looked for sports-related information or they received (understandable) information through school, local newspapers, or in their community. Another reason is that participants had no difficulty understanding or providing the information necessary for their children’s sports participation.

In sum, a number of participants experienced a misfit between the availability of information and their need for sports-related information. Others experienced a misfit between the information (systems) and their information capacities. Environmental attributes involved in this PE misfit were the lack of understandable information, too complex information systems, and a lack of support. Individual attributes were suboptimal skills in reading, writing, and in dealing with online media. The main misfit-reducing strategy deployed by participants was arranging support. Misfit was experienced to a lesser extent by participants who felt that understandable information was available (environmental attribute) or that they had sufficient information (seeking) skills (individual attribute).

### 3.5. Arranging and Accepting Support

As described in previous sections, participants mentioned that they arranged and/or accepted support from others outside their households to reduce misfits in relation to financing sports participation, transporting their children, and in understanding and providing information. Participants also stated that they experienced difficulties in arranging or accepting support. They mentioned that they had few/no family members, friends, or other individuals who could assist them in organizing sports participation. Some indicated that they did not know others well enough to ask for support:

“I don’t have many contacts. Once I know somebody, it’s okay. But like: “Hi, can you help me with bringing my child [..]?” I won’t ask someone I don’t know that. That’s only for good friends. (Interview 3).”

Others experienced rejection in response to requests for support (also in areas outside sports) or were treated disrespectfully by individuals in their environment, leading to their distrusting others and fearing rejection. Furthermore, participants experienced feelings of shame and failure for not being able to organize sports participation/raise children without support. Some participants, however, overcame their hesitation about arranging support in order to succeed in arranging the necessary support for sports participation:

“I can imagine that it’s hard to ask for help. But I prefer asking for help rather than having my child sitting at home without any sports. I think that’s worse.… I would do anything to make sure that my child participated in sports. (Interview 14).”

This illustrates a finding in multiple interviews, namely, that participants dealt with this misfit by prioritizing the child’s interest and sports participation over their own hesitation about arranging support.

No or little difficulty in arranging support was experienced by participants who had positive previous experiences and believed that asking for support was normal and common. Moreover, they had a social network consisting of individuals willing to provide support.

In general, the results show that multiple participants experienced a PE misfit between the support resources in their social environment and the capacities to arrange support. Environmental attributes involved in this misfit were a lack of trusted, supportive, and respectful individuals in their social environment. Individual attributes included shame and fear of rejection. Participants’ main strategy to deal with this misfit was to prioritize children’s sports participation over their own hesitation about arranging support. No or little difficulty in arranging support was experienced by participants who had a supportive social network (environmental attribute). Moreover, they had a positive attitude toward, and perceived norm in favor of, arranging support (individual attribute).

## 4. Discussion

Despite the many benefits of sports participation for children, studies in multiple countries have shown that sports participation is lower among children from low-income families than among children from middle- or high-income families (e.g., [4,5,6,7]). Parents play an important role in facilitating their children’s sports participation [3], but few studies to date have focused on understanding parent-related factors influencing children’s sports participation.

From a socioecological perspective, it is essential to increase the knowledge about the individual and environmental factors that impede or support children’s parents to facilitate sports participation, so that sports participation among children from low-income families can be increased. Therefore, the current qualitative study aimed to identify and describe low-income parents’ experiences of PE misfits during facilitation of children’s sports participation. The second aim of this study was to identify and describe parental strategies to reduce PE misfits, either by enhancing themselves or by enhancing their environment. Last, the aim was to describe experiences of parents who experienced no or little misfit.

Three main conclusions can be drawn from the results. The first main conclusion is that PE misfits were found in multiple behaviors related to the facilitation of children’s sports participation: financing sports participation; planning and investing time; transporting children; acquiring, processing, and providing information; and arranging support. These findings are consistent with the results of other studies showing that not only sports-related costs, but also transport and time, are experienced as barriers [13,14,15,16]. The current study adds to this knowledge that additional difficulties may be experienced by parents while acquiring, processing, and providing the information necessary to facilitate their children’s sports participation, for example, when registering at the sports clubs or acquiring financial support from fund providers. Furthermore, difficulties may be experienced in arranging support, such as for transport or for financing sports participation. Consequently, the findings of the current study encourage future studies and intervention for developers to look beyond costs when aiming to understand or enhance the facilitation of children’s sports participation by parents from low-income families.

The second main conclusion is that, across PE (mis)fits, influential attributes were found on the individual level as well as within multiple domains of the environmental level: the social (e.g., lack/presence of supportive network), policy (e.g., lack/presence of financially supportive policy of the municipality), physical (e.g., long or short distances), and information (e.g., lack/presence of understandable information) environment. Furthermore, it was found that these factors interact. To illustrate: we found that a long distance between the house and the sports club (physical environment) and/or the absence of transport (individual level) was experienced as less difficult by parents who had a supportive network for co-traveling (social environment) or who experienced no hesitation in arranging support for transport (individual level). Thus, the findings of this study are congruent with socioecological theories that suggest that behavior is influenced by individual as well as by environmental factors, and that factors interact across domains [17]. More specifically, these results point to the environmental domains in which relevant attributes may be located and how these attributes may interact. Future studies could aim to build upon these results by further exploring the relevance of the environmental and individual attributes that were found in relation to PE misfits, as well as the (influence of) interactions between these attributes.

The third main conclusion is that parents in response to experienced PE misfits deployed multiple strategies to reduce these PE misfits. They deployed strategies aiming to enhance themselves, such as increasing their financial capacities (e.g., by purchasing cheaper alternative sports clothing), increasing time capacities (e.g., by actively managing time), only choosing sports clubs nearby, and prioritizing their child’s sports participation. In addition, they deployed strategies with the aim of enhancing their environment, such as availing themselves of the financial support offered in their municipality and arranging social support. Furthermore, and in line with the socioecological perspective, these findings illustrate the dynamics of individual–environment relations and how individuals modify their environments in order to make their environments more supportive [19].

Overall, these results and the parents’ specific accounts of their experiences provide a detailed insight into PE misfits experienced during the facilitation of their children’s sports participation and into the environmental and individual attributes involved in these PE misfits. Thus, the current study supports the conclusion of other studies that socioecological theories provide a suitable framework for examining and understanding difficulties relating to parental facilitation of sports participation by children from low-income families [14,31]. The current study adds to this that applying the PE misfit concept may elucidate these difficulties by identifying the specific behaviors during which parents experienced difficulties. Knowing these specific behaviors is crucial, as socioecological models should be tailored to specific behaviors [17]. Furthermore, for effective intervention development, distinguishing between specific behaviors is vital: influential factors can differ between these behaviors and, in such cases, different intervention strategies are needed to modify these factors [32,33]. This also became evident in the results of the current study. For example, environmental and individual attributes involved in financing sports participation were different from those involved in transporting children. Therefore, different intervention strategies will be needed to reduce these different PE misfits.

Given that interventions to change behavior will be most impactful when interventions target behavioral determinants at multiple ecological levels and environments in a complementary and synergistic way [34,35], this study provides a set of promising and targeted entry points for multilevel interventions aimed at preventing or reducing specific PE misfits. For example, to prevent or reduce PE misfits in acquiring, understanding, and providing information, interventions focused on parents’ information environment could aim to increase the availability and user-friendliness of information (systems) on sports and financial support, or to change policies in such a way that complex procedures become redundant. Furthermore, interventions could focus on parents’ social environment by increasing social support for understanding information, for example within the neighborhood or at the sports club. At the same time, individual-focused interventions could concentrate on improving parental information and digital skills.

Although the study has provided important insights into parental facilitation of their children’s sports participation, some limitations should be addressed. First, despite different strategies being used to recruit participants, all participants were able to read information in Dutch and most of them had successfully applied for financial support from charitable organizations. Therefore, the study included a subgroup of low-income parents with Dutch language skills, the majority of whom went through an application process. Their skills, motivations, thoughts, and experiences may differ from those who are not able to read information in Dutch or have not succeeded in going through this application process. Potentially, these parents experience even more difficulties with, for example, financing sports participation or acquiring, processing, and providing information. If so, relevant individual and environmental attributes other than those found in the current study may be identified. Second, the vast majority of participants self-identified as female. Female parents’ thoughts and experiences potentially diverge from those of male parents, because male and female parents may play different roles in relation to the child’s upbringing [36]. The consequence of these two limitations is that potentially other factors not identified in this study may also be important or that factors work differently outside the current sample. Third, because only parents from low-income families were included in the study, no conclusions can be drawn about whether the identified PE misfits are unique to parents from low-income families. Costs and time have been found to be experienced as more of a barrier by parents from low-income families than by parents from non-low-income families [13]. The wider applicability of the other PE misfits found in this study remains unknown. To address this gap, future studies should include a group of parents from middle- and high-income families and compare the degree to which these groups experience the PE misfits identified in this study.

## 5. Conclusions

This study provides detailed insight into how the study sample of parents from low-income families experienced the facilitation of their children’s sports participation. PE misfits were found in multiple behaviors related to the facilitation of children’s sports participation: financing sports participation; planning and investing time; transporting children; acquiring, processing, and providing information; and arranging support. Across these PE (mis)fits, influential attributes were found on the individual level as well as within multiple domains of the environmental level (e.g., social and physical). In response to PE misfits experienced, parents deployed multiple strategies to reduce these PE misfits, aimed at enhancing either themselves or their environments. These results show the complexity of the factors that may influence the degree to which parents from low-income families are able to facilitate their children’s sports participation. Consequently, the results contribute to an awareness of the complexity and relevance of environmental and individual attributes among professionals and policymakers who aim to increase sports participation among children from low-income families. Moreover, the results may guide future research and the development and/or implementation of multilevel interventions.

## Data Availability

The data presented in this study are available on reasonable request from the corresponding author. The data are not publicly available to protect participants’ privacy.

## Supplementary Materials

The following supporting information can be download at: https://www.mdpi.com/article/10.3390/children9111746/s1, See Table S1 for information about the participants collected during the telephone conversations, the online surveys, and the interviews.

## References

[1] Patterson M.S., Amuta A.O., McKyer E., McWhinney S.L., Outley C.W., Tisone C.A. (2015). The physical activity environment among rural, low-income children. Health Behav. Policy Rev..

[2] World Health Organization (2011). Information Sheet: Global Recommendations on Physical Activity for Health for 5–17 Year Olds.

[3] Eime R.M., Young J.A., Harvey J.T., Charity M.J., Payne W.R. (2013). A systematic review of the psychological and social benefits of participation in sport for children and adolescents: Informing development of a conceptual model of health through sport. Int. J. Behav. Nutr. Phys. Act..

[4] Larocca V., Wilson S., Cavaliere A. (2018). Examining the association between parent and child sport participation in Canada: A general social survey study. Can. J. Fam. Youth.

[5] Clark W. (2008). Kids’ sports. Component of Statistics Canada Catalogue no. 11-008-X. Can. Soc. Trends.

[6] Kwon S., Janz K.F., Letuchy E.M., Burns T.L., Levy S.M. (2016). Parental characteristic patterns associated with maintaining healthy physical activity behavior during childhood and adolescence. Int. J. Behav. Nutr. Phys. Act..

[7] Tandon P.S., Kroshus E., Olsen K., Garrett K., Qu P., McCleery J. (2021). Socioeconomic inequities in youth participation in physical activity and sports. Int. J. Environ. Res. Public Health.

[8] Heijnen E., Dellas V., Elling A. (2020). Verschillen in Lidmaatschap Sportverenigingen Middelbare Scholieren Naar Opleiding, Etniciteit, Gezinswelvaart en Geslacht [Differences Is Sports Club Membership of High School Students by Education, Ethnicity, Family Affluence and Sex] (2005–2017).

[9] Kenniscentrum Sport & Bewegen [Knowledge Centre for Sport & Physical Activity] (2017). Jeugd-Armoede-Sport Feiten & Cijfers [Youth-Poverty-Sports Facts & Numbers].

[10] Somerset S., Hoare D.J. (2018). Barriers to voluntary participation in sport for children: A systematic review. BMC Pediatr..

[11] Rittsteiger L., Hinz T., Oriwol D., Wäsche H., Santos-Hövener C., Woll A. (2021). Sports participation of children and adolescents in Germany: Disentangling the influence of parental socioeconomic status. BMC Public Health.

[12] Dollman J., Lewis N.R. (2010). The impact of socioeconomic position on sport participation among South Australian youth. J. Sci. Med. Sport.

[13] Rodrigues D., Padez C., Machado-Rodrigues A.M. (2019). Parental perception of barriers to children’s participation in sports: Biological, social, and geographic correlates of Portuguese children. J. Phys. Act. Health.

[14] Holt N.L., Kingsley B.C., Tink L.N., Scherer J. (2011). Benefits and challenges associated with sport participation by children and parents from low-income families. Psychol. Sport Exerc..

[15] Kingsley B.C., Spencer-Cavaliere N. (2015). The exclusionary practices of youth sport. Soc. Incl..

[16] Hardy L.L., Kelly B., Chapman K., King L., Farrell L. (2009). Parental perceptions of barriers to children’s participation in organised sport in Australia. J. Paediatr. Child Health.

[17] Sallis J.F., Owen N., Fisher E., Glanz K., Rimer B.K., Viswanath K. (2008). Ecological Models of Health Behavior. Health Behavior: Theory, Research, and Practice.

[18] Gubbels J.S., Van Kann D.H., de Vries N.K., Thijs C., Kremers S.P. (2014). The next step in health behavior research: The need for ecological moderation analyses—An application to diet and physical activity at childcare. Int. J. Behav. Nutr. Phys. Act..

[19] Stokols D. (1996). Translating social ecological theory into guidelines for community health promotion. Am. J. Health Promot..

[20] Spence J.C., Lee R.E. (2003). Toward a comprehensive model of physical activity. Psychol. Sport Exerc..

[21] Kaplan S. (1983). A model of person–environment compatibility. Environ. Behav..

[22] Van Vianen A.E. (2018). Person–environment fit: A review of its basic tenets. Annu. Rev. Organ. Psychol. Organ. Behav..

[23] Saint-Onge K., Bernard P., Kingsbury C., Houle J. (2021). Older public housing tenants’ capabilities for physical activity described using walk-along interviews in Montreal, Canada. Int. J. Environ. Res. Public Health.

[24] Dunning T., Camp E. (2015). 0_Camp_Anonymization Protocol.pdf. Brokers, Voters, and Clientelism: The Puzzle of Distributive Politics.

[25] Annink A., Van Leeuwen L. Understanding parents’ and professionals’ experiences with organizing financial support for children’s club-organized sports participation. Manuscript under Review.

[26] QSR International Pty Ltd (2018). NVivo (Version 12). https://www.qsrinternational.com/nvivo-qualitative-data-analysis-software/home.

[27] Silverman D. (2017). Doing Qualitative Research.

[28] Hsieh H.-F., Shannon S.E. (2005). Three approaches to qualitative content analysis. Qual. Health Res..

[29] Hennink M., Kaiser B.N. (2021). Sample sizes for saturation in qualitative research: A systematic review of empirical tests. Soc. Sci. Med..

[30] Neale J., Miller P., West R. (2014). Reporting quantitative information in qualitative research: Guidance for authors and reviewers. Addiction.

[31] Harrington D.W., Jarvis J.W., Manson H. (2017). Parents’ perceived barriers to accessing sports and recreation facilities in Ontario, Canada: Exploring the relationships between income, neighbourhood deprivation, and community. Int. J. Environ. Res. Public Health.

[32] Peters G.-J. (2014). A practical guide to effective behavior change: How to identify what to change in the first place. Eur. Health Psychol..

[33] Eldredge L.K.B., Markham C.M., Ruiter R.A., Fernández M.E., Kok G., Parcel G.S. (2016). Planning Health Promotion Programs: An Intervention Mapping Approach.

[34] Sallis J.F., Cervero R.B., Ascher W., Henderson K.A., Kraft M.K., Kerr J. (2006). An ecological approach to creating active living communities. Annu. Rev. Public Health.

[35] Weiner B.J., Lewis M.A., Clauser S.B., Stitzenberg K.B. (2012). In search of synergy: Strategies for combining interventions at multiple levels. J. Natl. Cancer Inst. Monogr..

[36] Romani A.Q. (2020). Parental behaviour and children’s sports participation: Evidence from a Danish longitudinal school study. Sport Educ. Soc..

