# Supplementary material for: Facilitating Children’s Club-Organized Sports Participation: Person–Environment Misfits Experienced by Parents from Low-Income Families
Source: Children (Basel). 2022 Nov 14;9(11):1746. doi: 10.3390/children9111746 (PMC9688730; doi:10.3390/children9111746)
Supplement: Supplementary file 1 [file children-09-01746-s001.zip › children-1974162-supplementary.pdf]

Supplemental Materials

Table S1
Sample characteristics

**Characteristic**	**Frequency**	**Characteristic**	Frequency
Self-reported parental age (years)[a]		Parent-reported current sport status of child(ren) (4–18) in the family[b]	
18 or 19	0		
20–24	0	Sport participants only	12
25–29	1	Non-sport participants only	8
30–34	3	Both sport and non-sport participants	4
35–39	7	Parent-reported number of children in the family[b]	
40–44	5		
45–50	4	1	5
51+	6	2	3
Self-reported parental gender[a]		3	7
Female	22	4	8
Male	4	5+	1
Not female or male	0	Parent-reported ages of the children in the family[c]	
Both female and male	0		
Self-reported parental educational level[a]		0–4	8
		5–9	12
No education	0	10–14	30
Primary education	2	15–17	9
Pre-vocational secondary education	2	18+	8
		Missing	5
Senior general secondary education	2	Parent-reported gender of children in the family[c]	
Pre-university education	0	Male	43
Secondary vocational education and training	22	Female	23
		Missing	6
Higher vocational education	3		
University education	1		
Other	5		
Self-reported parental country of birth[a]			
The Netherlands	13		
Outside The Netherlands	13		

[a] N_{total} = 26 participants.
[b] N_{total} = 24 families.
[c] N_{total} = 72 children.
